# Prevalence, side effects and awareness about energy drinks among the female university students in Saudi Arabia

**DOI:** 10.12669/pjms.332.12084

**Published:** 2017

**Authors:** Mohamudha Parveen Rahamathulla

**Affiliations:** 1 Dr. Mohamudha Parveen Rahamathulla, PhD, Department of Medical Lab Sciences, College of Applied Medical Sciences, Prince Sattam bin Abdulaziz University, Wadi Al Dawaser-11991, Kingdom of Saudi Arabia

**Keywords:** Adverse effects, Awareness, Consumption, Energy Drink, Prevalence

## Abstract

**Objective:**

To evaluate the consumption, prevalence, side effects and awareness of energy drinks among female university students in Saudi Arabia.

**Methods:**

A quantitative research design was implied with sample size of 358 female students, recruited from Prince Sattam bin Abdulaziz University. The data, gathered through self-administered questionnaire, was analyzed through SPSS version 20.0 with p value <0.005 deemed statistically significant.

**Results:**

From the sample of 358 female students, 337 attempted the questionnaire from which 274 students were identified as energy drink consumers. The reasons for increased consumption of energy drinks mainly include giving company to friends (59.4%), better performance in exams (41.2%), and better concentration in studies (39.4%). The most common side effect was headache (32.3%), and the least was identified as allergy (2%). Only 39.4% and 29.9% of students acquired awareness regarding the harmful effects of energy drink consumption during pregnancy and breast feeding respectively.

**Conclusion:**

A significant proportion of female students at Prince Sattam bin Abdulaziz have reported to consume energy drinks regularly with several adverse effects. The government of Saudi Arabia should take serious initiatives towards organizing effective awareness programs specifically in universities and colleges to control the consumption of energy drinks and educate on the adverse effects.

## INTRODUCTION

The consumption of energy drinks is a common trend among youth, which is increasing at a constant rate. Taurine, carbohydrates, caffeine, glucuronolactone, and patanol are common ingredients, which are used to prepare energy drinks. Vitamin B and proprietary substances are also used in energy drinks.^1^ In the European markets; the demand for energy drinks has been increased for more than a decade. However, the energy drinks were banned in Turkey due to presence of higher level of caffeine. The effects were found due to high content of caffeine in energy drinks, which included increase in stamina and alertness that is attractive for the young people or students going to work or college. The excessive amount of caffeine in energy drinks can work as an addictive substance or sometime can also cause intoxication.^2^ It has been reported that caffeine is the chief ingredient present in drinks acting as a stimulant, which can affect the central nervous system (CNS). Such impairment can also result in the activation of sympathetic adrenal-medullar system.^3^ A research investigated that a 100 ml of energy drink contains 80-242 mg of caffeine that is equivalent to consumption of eight cups of strong coffee in a day.^4^ The overdose of caffeine can also cause greater risk of toxicity and disturb the overall health structure particularly among youngsters.

One of the studies have reported that regular consumption of energy drinks can lead to enhanced mental and physical performance of young people.^5^ Across the world, the consumption and supply of energy drinks have increased extensively. In 1997, the energy drinks were initially introduced in the market with the launch of Red Bull, which ultimately was followed by different trade names. The younger generation tends to consume the energy drinks in order to showcase their modern and active lifestyle; whereas, rest of the consumers purposely consume it for increasing their cognitive functioning earlier to attempt a certain task. In college and university students, the consumption of energy drinks has been found at a higher rate, specifically during exams or presentations.^6^ Among some of the famous countries, Saudi Arabia has marketed energy drinks aggressively from the last two decades. Above 500 new drinks were launched in 2006 across the globe that led the beverages companies procuring revenue around $5.7 billion in the energy drinks industry.^7^

According to another study, the consumption of energy drinks in the college students was investigated, and found a greater prevalence of consumption in the female students of a medical college along with excessive use of sugar free energy drinks varieties.^8^ Yet another study conducted in Turkey also reported 32.5% prevalence of energy drink consumption in a medical school.^2^ The similar study also stated that both the sexes were highly addicted to consume the energy drinks, and no considerable differences were found. Furthermore, a study investigated that 51% and 48.3% of students felt highly attracted towards consuming energy drinks.^9^ The rate of prevalence related with energy drink consumption in medical college students to those of athlete college students was found to have a substantial difference. With a prevalence of 62.2% among the athlete students.^10^ Similarly, athletes preferred to consume energy drinks to improve their physical performance; therefore, they were more inclined towards the consumption of energy drinks as compared to other students.

Apart from increasing the attraction, alertness and stamina among young individuals, energy drinks can also effect negatively. These negative effects are mostly associated with the toxicity of its constituents or over consumption. Therefore, it has been suggested that caution notice is a necessary element, which must be printed on the packaging of energy drinks for making the young consumers aware regarding benefits and adverse effects.^11^ Certain common side effects mainly include increased diuresis, insulin resistance, and increased blood pressure.^6^ Additionally, the nervousness irritability, tremors, muscle twitching, disturbed sensation, increasing palpitation, arrhythmias, gastro-intestinal disturbance and increasing depression are also the associated side effects.^8^

### Research Problem

According to Global Energy Drinks Report,^12^ specific warnings were issued regarding the Kingdom of Saudi Arabia and declared as one of the top ten nations, where citizens consumed excessive energy drinks.^12^ After crucial warning, the Kingdom of Saudi Arabia took strict actions to ban the sale of energy drinks within the colleges, universities and government premises. Moreover, the aggressive marketing and advertisements were also banned by the government authorities and restrictions were imposed on the print and electronic media to advertise any energy drink product.^13^

The research in this study indicated the availability of energy drinks in markets, and café of colleges and universities, where no proper information was provided for the adverse effects. Therefore, a great concern exists regarding the consumption of energy drink within the younger generation. Further research is required in the domain, to identify the protection and safety profile for young generation. It is important to provide proper education over the harmful effects of excessive energy drink intake, and their serious medical problems in the students specifically. The previous studies conducted on this subject mostly aimed at investigating medical or other students entailing both genders; however, no major study has been conducted highlighting energy drink consumption by females peculiarly.

## METHODS

The cross sectional survey has recruited 358 female university students from Prince Sattam bin Abdulaziz. The study was accomplished within time duration of 3 months, from December 2015 to February 2016. The university is located in Wadi Al Dawaser of Riyadh province, which is the eastern region of Saudi Arabia. Students from all over the country are enrolled in this university.

Quantitative approach followed the trend of random convenience sampling, was utilized to recruit 358 female students. Moreover, a multi-stage sampling technique was implemented that included 3^rd^, 4^th^, and 5^th^ years students. A self-administered questionnaire in English and Arabic Language was generated for data collection. The items in the questionnaire were kept simple for the convenience of parents and guardian. The questionnaire assessed socio-demographics, consumption habits, experiences of side effects and awareness of energy drinks.

The data collected through the questionnaire were pre-tested on 10 students to ensure the readability and precise administration of data collection forms. In order to analyze the collected data, it was entered into SPSS software version 20.0. Chi-Square test was applied to test the association among the variables and p value < 0.005 was considered statistically significant with 95% confidence level. Before the initiation of the study, approval was taken from ethics committee of the university. Moreover, proper consent was obtained from each participant, who were included in the research. For maintaining the ethics, the confidentiality of the participants was kept secured throughout the study.

## RESULTS

From a total of 358 female participants, 337 responded to the questionnaire with keen interest, while the rest did not complete the questionnaire. The response rate generated for 337 responses was 94.1%. A total of 274 with response rate 81.3% were identified as regular energy drink consumers along with the responses for non-consumers (19%).

**Fig.1:**
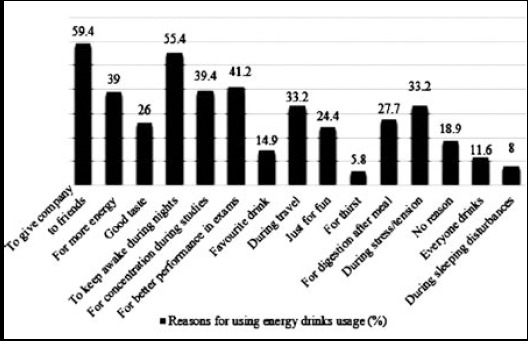
Prevalent reasons for energy drinks usage among female students.

The results highlighted that 126 students preferred recreational places for consuming energy drinks, 53 students consumed drinks during travelling, 48 students preferred consumption at home, and 47 students reported other places. Fig.1 has highlighted a range of reasons that promote consumption of energy drinks, where, 163 students indicated that they consumed energy drink to accompany friends, 113 students indicated for better performance in exams, and 108 students reported for concentration during studies. Fig.1 further shows that consumption habit of energy drinks increases in social circle that make them feel active around.

Eighty one students reported that, they started consuming energy drinks between ages of 10-12 years; however, other 127 students reported that they started to consume energy drinks at the age of 16 years and above. It has shown that majority of the female students started consuming energy drink at age above 16 years. Ninety seven students stated, that they heard from their friend first time about energy drink, 43 students stated that they heard through food labels, and 31 stated internet sources. The response rates of 35.4%, 15.6% and 11.3% respectively.

One hundred eighty two students preferred to choose regular drink, 39 students preferred sugar free drink and 53 students preferred both regular or sugar free drinks. Flavor of the drink was identified to be a significant aspect while purchasing. Majority of the students (163) reported that flavor is the most essential part to purchase energy drinks. About the consumption per day, 143 students reported that they consume one drink per day, and 112 reported that they prefer during the meal time. Findings demonstrate, that drinking on daily basis is comparatively higher.

**Fig.2:**
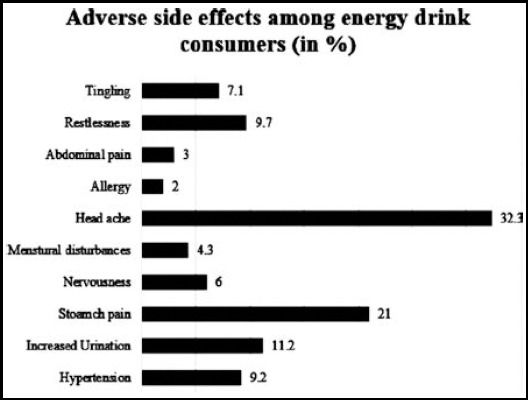
Percentage of adverse side effects experienced among energy drink consumers.

Among several potential benefits of consuming energy drink, ability to stay awake for longer time was stated by majority of the students. One hundred and sixteen students stated increased concentration as a major reason for consumption, and 81 students responded for alertness. Twenty six students indicated that there were no benefits with the consumption of energy drink. The adverse side effects were reported in 195 students, who had noticed one or more side effects after consuming it. Sixty three of them reported headache, 41 suffered from stomachache, 22 reported frequent urination, and 12 females reported disturbance in periodic cycle. Fig.2.

## Knowledge about Usage of Energy Drinks

**Fig.3:**
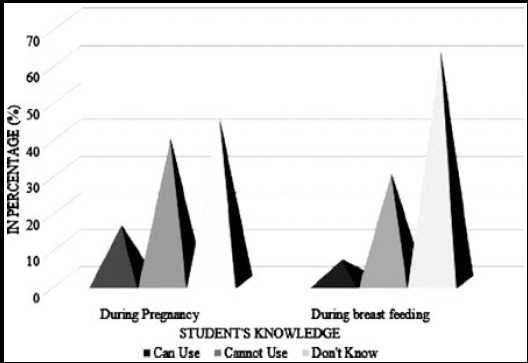
Knowledge about usage of energy drinks among female students.

From a total of 274 energy drink consumers, 163 students reported as not to recommend the energy drinks to others. Eighty six students reported that energy drinks are harmful for the health, 52 reported they do not know, and 136 students reported they do not have ample knowledge regarding the usage. Apart from knowing the effects of energy drinks, 108 students stated that they were aware about the ingredient present in the drinks which were harmful during pregnancy. On the other hand, 82 reported that they were aware about some ingredients, which are harmful during breast feeding. Fig.3 represents knowledge regarding the consumption of energy drinks. However, most of the students were unaware about the harmful ingredients.

As regards the choice of brands preferred by the students, majority of them agreed on choosing Code Red; however, Bison acquired the second position in brand preferences as shown in Table-I. Red Bull, despite of being the first energy drink, acquired third position in brand preferences. Through the questionnaire, female students demonstrated several different reasons for consuming energy drinks and manifested side effects as a result of consumption. The least side effects evaluated were allergy and abdominal pain with 2% and 3% respectively. Headache was found as the major side effect with 32.3%. Table-I

**Table-I:** Energy drinks preferred among University female students.

*Brand of the Drink*	*No. of Consumers (n=274)*	*(%)*
Code Red	62	22.6
Bison	53	19.3
Red Bull	46	16.7
Holsten	37	13.5
Power Horse	18	6.5
Dandanah	12	4.3
Perriea	9	3.2
Black	9	3.2
Real Madrid	6	2.1
Others	22	8

## DISCUSSION

The expansion in energy drink market over the last couple of years has been observed due to non-existence of clear regulations. This research was aimed at evaluating the consumption of energy drinks among female students within Prince Sattam bin Abdulaziz University. Most of the students do not have any specific reasons for using energy drink for the first time. Company of friends deemed to be a common reason for consuming energy drink for the first time.^14^ The present study has also reported company of friends (59.4%) as most common reason for energy drinks usage among female students.

A study reported that 29% of the participants experienced crash episode, 22% experienced severe headache and 19% suffered from heart palpitations while consuming the energy drinks.^8^ Another study reported that consumers suffer for short term illnesses from which they are usually not aware that can lead to severe health issues in long term including depression, heart palpitations, and nausea.^12^ Additionally, the results have also supported the fact of high percentage of headache (32.3%) as a side effect faced by the female students. Furthermore, a study also indicated increased urination and insomnia as the most common adverse effects reported by the students consuming energy drinks, regularly.^15^

Among other elements, one significant achievement of this study was to understand the underlying reasons of female consumption of energy drinks. However, female students indicated better performance in exams more frequently as compared to male students.^16^ The results also highlighted that females preferred energy drinks for performing better in their examinations that was estimated about 41.2% and the least among was for thirst. Conversely, a research conducted in Argentina evaluated that 54% of participants used energy drinks to improve their taste buds, 27.7% consumed it as a leisure activity, and 4.4% used during learning processes.^17^ Similar results were recorded in another study conducted in Pennsylvania, where only 20% from the total population stated the reason for consumption, which was to stay awake for late hours to study better.^18^

According to another study, there were no adverse effects associated with substances like taurine, guarana, and ginseng, but the content of caffeine in energy drink has major side effects such as insomnia, hypertension, headache, and tachycardia.^19^ Moreover, the consumption of energy drinks excessively leads consumers towards toxic addiction. This toxic addiction in long term can turn into several health issues such as depression, arterial blood pressure, and heart palpitation.

The increase in the consumption of energy drinks has shown that it also has effect on the monthly income of consumers ultimately influencing their consumption patterns. One of the major concerns for consuming the energy drinks is that it can extend the drinking session for longer time period.^20^ Thus, regulatory actions are required to reduce the level of caffeine in energy drinks, and future studies should be conducted on the risk associated with over consumption. Furthermore, the consumption of energy drinks effects the nutrition and health, with a negative effect on the metabolism leading to health concerns. A study has also stated some of the risk factors for excessively consuming energy drinks that are related to greater risk of obesity, high triglyceride, high density lipoprotein cholesterol, and increasing glucose level. These risk factors are collectively known as metabolic syndrome that a person experience at an early age and thus metabolic risk can lead to higher obesity risk, diabetes mellitus, and blood pressure.^21^ Therefore, higher consumption of energy drinks are also associated to risk factors of generating strong traits of metabolic syndrome.

## CONCLUSION

The consumption of energy drink is a common practice among the students and young people. The prevalence of energy drink consumption was studied within the female university students, and found that 274 students agreed on energy drink consumption. The Government of Saudi Arabia should take major steps to control its sale and aggressive marketing of energy drinks. The study has further highlighted various reasons for energy drink consumption. The prevalence of various energy drink is on the increase, and the participants also manifested various side effects. Headache and palpitation were the most frequently reported side effects. Apart from the prevalence and adverse effects of energy drinks, the students did not have awareness towards the energy drink usage, effects, benefits, and basic information. It is recommended that public education and awareness programs regarding the potential benefits and adverse effects may be arranged. Future research should be conducted to understand the risk and possible interventions for stimulating protective consumption of energy drinks.

## References

[1] O'Brien MC, McCoy TP, Rhodes SD, Wagoner A, Wolfson M (2008). Caffeinated cocktails:energy drink consumption, high-risk drinking, and alcohol-related consequences among college students. Acad Emerg Med.

[2] Hidiroglu S, Tanriover O, Unaldi S, Sulun S, Karavus M (2013). A survey of energy-drink consumption among medical students. J Pak Med Assoc.

[3] Lane JD, Pieper CF, Phillips-Bute BG, Bryant JE, Kuhn CM (2002). Caffeine affects cardiovascular and neuroendocrine activation at work and home. Psychosom Med.

[4] McCusker RR, Goldberger BA, Cone EJ (2006). Caffeine content of energy drinks, carbonated sodas, and other beverages. J Anal Toxicol.

[5] Higgins JP, Tuttle TD, Higgins CL (2010). Energy beverages:content and safety. Mayo Clin Proc.

[6] Bawazeer NA, AlSobahi NA (2013). Prevalence and side effects of energy drink consumption among medical students at Umm Al-Qura University, Saudi Arabia. Inter J Med Students.

[7] Boyle M (2006). Monster on the Loose-Fueled by its energy drink, Hansen Natural is on a rocket ride. Is the fast-grower due for a crash?. Fortune.

[8] Malinauskas BM, Aeby VG, Overton RF, Carpenter-Aeby T, Barber-Heidal K (2007). A survey of energy drink consumption patterns among college students. Nutr J.

[9] Pettit ML, DeBarr KA (2011). Perceived stress, energy drink consumption, and academic performance among college students. J Am Coll Health.

[10] Paddock R (2008). Energy drinks'effects on student-athletes and implications for athletic departments. Sport J.

[11] O'Dea JA (2003). Consumption of nutritional supplements among adolescents:usage and perceived benefits. Health Educ Res.

[12] (2012). Global Energy drink report.

[13] Naeem Z (2014). Health hazards of Energy Drinks and positive actions by Saudi Government. Intern J Health Sci.

[14] Attila S, Çakir B (2011). Energy-drink consumption in college students and associated factors. Nutrition.

[15] Alsunni AA, Badar A (2011). Energy drinks consumption pattern, perceived benefits and associated adverse effects amongst students of University of Dammam, Saudi Arabia. J Ayub Med Coll Abbottabad.

[16] Miller KE (2008). Energy drinks, race, and problem behaviors among college students. J Adolesc Health.

[17] Ballistreri MC, Corradi-Webster CM (2008). Consumption of energy drinks among physical education students. Rev Lat Am Enfermagem.

[18] Clauson KA, Shields KM, McQueen CE, Persad N (2008). Safety issues associated with commercially available energy drinks. Pharm Today.

[19] Ragsdale FR, Gronli TD, Batool N, Haight N, Mehaffey A, McMahon EC (2010). Effect of Red Bull energy drink on cardiovascular and renal function. Amino acids.

[20] Hidiroglu S, Tanriover O, Unaldi S, Sulun S, Karavus M (2013). A survey of energy-drink consumption among medical students. J Pak Med Assoc.

[21] Dhingra R, Sullivan L, Jacques PF, Wang TJ, Fox CS, Meigs JB (2007). Soft drink consumption and risk of developing cardiometabolic risk factors and the metabolic syndrome in middle-aged adults in the community. Circulation.

